# Immune Thrombocytopenia (ITP): Current Limitations in Patient Management

**DOI:** 10.3390/medicina56120667

**Published:** 2020-11-30

**Authors:** Deirdra R. Terrell, Cindy E. Neunert, Nichola Cooper, Katja M. Heitink-Pollé, Caroline Kruse, Paul Imbach, Thomas Kühne, Waleed Ghanima

**Affiliations:** 1 Department of Biostatistics and Epidemiology, Hudson College of Public Health, University of Oklahoma Health Sciences Center, Oklahoma City, OK 73104, USA; 2 Department of Pediatrics, Columbia University Irving Medical Center, New York, NY 10032, USA; cn2401@cumc.columbia.edu; 3 Centre for Haematology, Department of Medicine, Hammersmith Hospital, Imperial College London, London SW7 2BU, UK; n.cooper@imperial.ac.uk; 4 Department of Pediatric Hemato-oncology, Princess Maxima Center, 3584 Utrecht, The Netherlands; k.heitink-polle@prinsesmaximacentrum.nl; 5 Platelet Disorder Support Association, Cleveland, OH 44141, USA; ckruse@pdsa.org; 6 Medical Faculty, University of Basel, 4051 Basel, Switzerland; paul.imbach@unibas.ch; 7 University Children’s Hospital, Oncology/Hematology, 4056 Basel, Switzerland; thomas.kuehne@ukbb.ch; 8 Departments of Hemato-oncology and Research, Østfold Hospital, 1714 Grålum, Norway; Wghanima@gmail.com; 9 Department of Hematology, Oslo University Hospital and Institute of Clinical Medicine, University of Oslo, 0318 Oslo, Norway

**Keywords:** immune thrombocytopenia (ITP), quality of life, treatment, refractory

## Abstract

Primary immune thrombocytopenia (ITP) is an acquired autoimmune disorder characterized by isolated thrombocytopenia caused by increased platelet destruction and impaired platelet production. First-line therapies include corticosteroids, intravenous immunoglobulin, and anti-D immunoglobulin. For patients who are refractory to these therapies, those who become corticosteroid dependent, or relapse following treatment with corticosteroid, options include splenectomy, rituximab, and thrombopoietin-receptor agonists, alongside a variety of additional immunosuppressive and experimental therapies. Despite recent advances in the management of ITP, many areas need further research. Although it is recognized that an assessment of patient-reported outcomes in ITP is valuable to understand and guide treatment, these measures are not routinely measured in the clinical setting. Consequently, although corticosteroids are first-line therapies for both children and adults, there are no data to suggest that corticosteroids improve health-related quality of life or other patient-related outcomes in either children or adults. In fact, long courses of corticosteroids, in either children or adults, may have a negative impact on a patient’s health-related quality of life, secondary to the impact on sleep disturbance, weight gain, and mental health. In adults, additional therapies may be needed to treat overt hemorrhage, but unfortunately the results are transient for the majority of patients. Therefore, there is a need to recognize the limitations of current existing therapies and evaluate new approaches, such as individualized treatment based on the probability of response and the size of effect on the patient’s most bothersome symptoms and risk of adverse effects or complications. Finally, a validated screening tool that identifies clinically significant patient-reported outcomes in routine clinical practice would help both patients and physicians to effectively follow a patient’s health beyond simply treating the laboratory findings and physical symptoms of ITP. The goal of this narrative review is to discuss management of newly diagnosed and refractory patients with ITP, with a focus on the limitations of current therapies from the patient’s perspective.

## 1. Introduction

Primary immune thrombocytopenia (ITP) is an acquired autoimmune disorder characterized by isolated thrombocytopenia caused by increased platelet destruction and impaired platelet production [1]. There is no definitive diagnostic test for ITP; therefore, primary ITP remains a diagnosis of exclusion after ruling out any underlying and/or initiating causes of the thrombocytopenia [2]. ITP is classified based on duration into newly diagnosed, persistent (3– <12 months), and chronic (≥12 months) [2]. ITP usually has a chronic course in adults [3], whereas approximately 80–90% of children undergo spontaneous remission within weeks to months of disease onset [4].

The goal of therapy is to cease any active bleeding and prevent future hemorrhage. The current American Society of Hematology clinical guidelines recommend managing a newly diagnosed adult patient with a platelet count <30 × 10^9^/L with corticosteroids with the addition of intravenous immunoglobulin (IVIG) in patients with active bleeding or those in whom a prompt increase in the platelet count is preferred [1]. In patients who do not respond adequately to first-line therapy, treatment options include thrombopoietin-receptor agonists (TPO-RAs), immune-modulators such as rituximab, or splenectomy [1]. Unlike adults, the recommendation for children with newly diagnosed disease who present with no or minor bleeding is observation, regardless of the platelet count. For children who present with bleeding and/or diminished health-related quality of life (HRQoL) the treatment recommendation is prednisone; however, depending on the severity of bleeding and need for a more rapid increase in platelet count, may include IVIG or anti-D immunoglobulin [1]. Yet, the signs and symptoms of ITP are highly heterogeneous and do not always correlate with the platelet count; consequently, the goal is a treatment plan tailored to the individual patient. This is especially important when the goal of treatment is to improve HRQoL or when treatment options vary considerably in the route of administration, side effects, or potential benefits. Current clinical guidelines recommend treatment decisions based on shared decision-making between the physician and patient, recognizing that each individual patient’s concerns, values, and preferences regarding treatment differ [1].

Shared decision-making can only occur however, if we can truly understand the challenges of living with ITP and use this information to guide treatment. It is therefore essential that physicians recognize what is most important about both the disease and the treatment from the patient’s perspective. Studies showed that in addition to bleeding risks, patients with ITP suffer from fatigue [5], impaired HRQoL [6], and often isolate themselves from social activities, which can result in depression [7]. Additionally, while the frequency of bleeding symptoms in patients with ITP is relatively low, the concerns over the potential for bleeding impose a psychological burden on the patient and interfere with work and family or social activities [8]. In fact, lower platelet counts are consistently associated with worse HRQoL [9]. The ITP World Impact Survey (I-WISh) survey, a cross-sectional survey of 1507 patients with ITP across 13 countries, found patients’ top three goals for ITP treatment were 1) achieving healthy platelet counts (64%), 2) preventing episodes of ITP from worsening (44%), and 3) increasing energy levels (41%) [10].

The goal of this narrative review is to discuss the management of newly diagnosed and refractory patients with ITP, with a focus on the limitations of current therapies from the patient’s perspective.

## 2. Limitations of Patient-Reported Outcomes in ITP

While many physicians focus on addressing low platelet counts as the best approach to manage their patients and avoid life-threatening bleeds, for many patients, the focus solely on platelet counts does not truly capture their experience with ITP. Instead, patients focus on the ways in which ITP negatively affects their lives, specifically how they feel and function. Fatigue is an important component of HRQoL in many chronic diseases. Patients with ITP cite fatigue as one of the most debilitating aspects of their condition, along with anxiety, fear, and frustration [10]. Patients with ITP also describe the belief that their fatigue severity correlates with decreased platelet counts [5]. Additionally, a recent study reported that fatigue severity does not improve in patients over time [11]. Yet the impact of fatigue on HRQoL is often ignored or underestimated by physicians, perhaps because fatigue is challenging for many reasons, including (1) fatigue is a common symptom that healthy and chronically ill individuals often report, (2) there is a lack of robust data regarding the extent of fatigue in ITP, (3) there is a lack of evidence regarding the effect of therapy on improving fatigue, and (4) the underlying pathophysiologic nature of fatigue in ITP remains unknown. As a result, the systematic assessment of fatigue is not included in the clinical evaluation and follow-up of ITP patients. However, for many patients with ITP, these symptoms are front and center among their concerns, rather than the objective measure of platelet counts. 

An assessment of patient-reported outcomes in ITP is valuable to understand and guide treatment [12]. Patient-reported outcomes are direct responses from patients about how they feel or function in relation to a health condition without interpretation by healthcare professionals. In ITP, there are valid, reliable instruments that exist to capture ITP disease-specific outcomes. These instruments include the ITP Patient Assessment Questionnaire (PAQ) [13] for adults and the Kids’ ITP Tools (KIT) [14] for children. The PAQ is a 44-item questionnaire that takes the patient approximately 10 to 15 min to complete. The PAQ includes six scales, namely, physical health (including fatigue/sleep), emotional health, social activity, work, women’s reproductive health, and overall quality of life [13]. In therapeutic ITP clinical trials, the PAQ is routinely administered to evaluate HRQoL, as it is able to detect clinically important changes related to fatigue, bother, and activity and also has acceptable reliability and validity [13,15,16]. The KIT is comprised of three questionnaires, i.e., one version for the child (aged 7–17 years of age), one version for the parent proxy to complete on behalf of the child (child aged 2–17 years of age), and one for the parent to complete which measures the impact of the disease on the parent [14]. The KIT was cross-culturally translated and is valid and reliable and used to assess HRQoL in clinical trials of children with ITP in the United States, France, Germany, the United Kingdom, and Uruguay [14,17]. 

Despite the availability of disease-specific tools, the use of patient-reported outcome instruments is limited to ITP research settings instead of clinical practice. Although clinicians state patient-reported outcomes are important to measure in order to understand the true burden of disease, they are concerned with the ability of a seamless integration of these instruments into the clinical workflow. Specifically, healthcare providers state concerns regarding burdening their staff with additional paperwork, increasing administrative costs, challenges with the clinical interpretation of the results of the instruments, and the potential for increased burden on patients [12]. Overcoming current limitations requires the development of instruments that are valid, yet simple to administer, score, and interpret in the clinical setting. 

## 3. Limitations of Current Therapies for Patients with Newly Diagnosed ITP

As mentioned above, newly diagnosed ITP patients often require treatment for various reasons. First, some patients present with significant hemorrhage that requires prompt treatment aimed at increasing the platelet count and a cessation of bleeding. Alternatively, the goal of treatment may be to increase the platelet count above a certain threshold given the concerns that more significant bleeding could occur in the setting of persistent severe thrombocytopenia [18]. The International Consensus Report guidelines recommend treatment should be administered to attain a minimum platelet count of 20–30 × 10^9^/L [2]. An additional reason for treatment, as outlined above, is to improve a patient’s overall HRQoL [1]. Improvements in the platelet count could result in improved HRQoL through minimizing the patient’s concern and fear of bleeding, reducing fatigue, and increasing ability to be involved in activities [9]. However, studies in both children and adults described that even in patients with raised platelet counts after treatment, fatigue (a component of HRQoL) was not always alleviated [4,8], which suggests that fatigue does not correlate with platelet count. A final rationale for providing treatment upfront is to reduce the likelihood of a patient developing chronic disease via early attempts at immunomodulation. However, there is a lack of evidence to suggest that the use of medications upfront in adult patients with ITP would prevent the development of chronic disease.

In children in whom treatment is necessary, the American Society of Hematology guidelines recommend a very short course of prednisone (<7 days) given the high likelihood of spontaneous remission and the desire to avoid side effects [1]. For children who need alternative therapies, options include IVIG and anti-D immunoglobulin. A systematic review of previous studies suggested that treatment with IVIG could prevent chronic ITP in children [19,20,21]. Unfortunately, these findings were not confirmed in a recent randomized trial, which reported no significant impact on chronicity at 6 or 12 months following IVIG administration at diagnosis [4]. In this same randomized trial, eight patients from the observation group required admission for nonfatal major bleeding in the first month after treatment [4]. However, there was one patient in the observation group who had spontaneous intracranial hemorrhage. The majority of the patients who had nonfatal bleeding (88%) had moderate (Grade 3) bleeding at the time of randomization [4], so in many clinical circumstances would have received treatment. This indicates that, for the majority of children with no or mild bleeding (< Grade 3), IVIG at the onset of ITP does not reduce bleeding; however, IVIG may be important in the management of Grade 3 or higher bleeding at the time of diagnosis.

In adults with ITP, traditional first-line therapy is oral corticosteroids. Many guidelines recommend limiting the use of corticosteroids to a maximum of six to eight weeks including the treatment dosing and taper, because higher dose corticosteroids given over a longer period may be harmful [1,2]. However, it remains unknown whether low-dose prednisolone can be safely used over a longer period if needed when it is well-tolerated. Oral corticosteroids initial response rates range from 70–80%; however, relapse rates are high, with low long-term remission rates [1]. Furthermore, long-term response outcomes do not appear to be different between regimens utilizing either dexamethasone (40 mg daily for four days) or prednisone (0.5–2 mg/kg/day over several weeks). In fact, results from a systematic review revealed no significant difference on overall (>30 × 10^9^/L) or complete (>100 × 10^9^/L) response to steroids at six months [22]. There are no robust predictors of response [23], therefore, physicians should consider a case-by-case determination of whether to use high-dose dexamethasone or prednisone based on individual patient characteristics and shared decision-making (i.e., side effect profiles for each regimen, adherence, or need for a more rapid response), recognizing that neither provides a long-term response in the majority of patients.

For adults who need alternative therapies, options also include IVIG and anti-D immunoglobulin. Generally, it is believed that treatment with either medication results in a more rapid increase in the platelet count compared to corticosteroids. It is important to note that both IVIG and anti-D immunoglobulin have significant side effect profiles. Patients receiving IVIG are at risk for allergic reactions, nausea/emesis, headache, aseptic meningitis, and renal failure requiring readmission [4]. The major concern with the use of anti-D immunoglobulin is reports of severe intravascular hemolysis, reported to be approximately 1 in 1000 [24,25].

Related to HRQoL, there are no data to suggest that corticosteroids improve HRQoL in either children or adults. In fact, long courses of corticosteroids, in either children or adults, may have a negative impact on HRQoL secondary to the impact on sleep disturbance, weight gain, and mental health [26]. Also, these patients may suffer from hypertension and diabetes while on corticosteroids which would then result in additional medications. Furthermore, the impact of IVIG or anti-D immunoglobulin on HRQoL is not well-studied.

## 4. Can We Improve Management?

While the above therapies may achieve the goals of treating overt hemorrhage, the results are transient for the majority of patients. The treatment goal of inducing an early remission (i.e., sustained elevation or normalization of platelet count without need for continuing therapy) is significant, as it limits the risk of severe bleeding and the need for additional therapies. Additionally, an early remission also results in a patient’s reduced exposure to treatment side effects, reduced treatment costs, and may improve HRQoL. Patients who fail to respond or become dependent on corticosteroids are usually offered one of the following second-line therapies: splenectomy, rituximab, or TPO-RA. The 2019 American Society of Hematology guidelines encourage shared decision-making with patients and a full assessment of the patient’s goals, lifestyle, preferences, and comorbidities, which may favor one therapy over another when evaluating treatment with splenectomy, rituximab, or TPO-RA [1]. Splenectomy is historically the treatment of choice of patients who are treatment-refractory to first-line therapy, although this is now used with much less frequency. Rituximab and TPO-RAs each provide an option for patients to either delay or potentially avoid splenectomy [1]. 

Rituximab is a monoclonal CD20 antibody that causes peripheral B-cell depletion. Randomized trials investigated dexamethasone in combination with rituximab versus dexamethasone alone in adult patients with treatment-naive ITP. Results from two studies demonstrated sustained response (platelet count >50 × 10^9^/L) in 58–63% of patients with combination therapy at six months compared to approximately 35% sustained response with dexamethasone use alone [27,28]. Gudbrandsdottir et al. reported a 12-month response of 53% in those receiving combination therapy compared to a 12-month response of 33% in the dexamethasone arm (*p* < 0.05) [28]. The side effect profile associated with rituximab treatment includes risk of infection associated with B-cell depletion, infusion reactions, and impaired response to vaccinations [2]. Although the combination of dexamethasone and rituximab as an upfront therapy is well documented, the use of this combination as an upfront therapy is not widely accepted, practiced, or recommended [1].

TPO-RAs act by stimulating platelet production. Currently, three TPO-RAs are -approved by the United States Food and Drug Administration for use in ITP, namely, eltrombopag, avatrombopag and romiplostim. There are limited published data on the effect of these agents on newly diagnosed patients. As a combination for intensified first-line therapy, a single-arm study with dexamethasone followed by a 28-day course of eltrombopag in treatment-naïve subjects showed 83% of patients had an early response and 50% of patients had a complete response at six months [29]. Simultaneous combination of eltrombopag and high-dose dexamethasone therapy could generate a sustained, complete, long-term response in adult patients and be used as a viable first-line treatment [30]. TPO-RAs are not universally considered curative, although in one study, 30% of adult patients with ITP maintained platelet counts >50 × 10^9^/L after discontinuation of romiplostim [31]. Further data are needed to understand how to utilize them in newly diagnosed patients and if the observed treatment-free responses truly represent a drug effect or the natural history of the disease in a select group of patients.

To improve management of ITP, we should move from treating all patients in the same way to more individualized treatment based on the probability of response and the size of effect on the patient’s most bothersome symptoms and risk of adverse effects or complications. Currently, we do not have the tools to adequately predict the risk of bleeding, disease course, or response/adverse effects to therapy, however, research in this area is ongoing. Regarding the risk of bleeding, early evidence suggests that flow cytometry-based platelet function is correlated with bleeding tendency in children as well as adults with ITP [32], but these tests are not regularly available for routine clinical use. Regarding disease course in children, studies showed there are some known risk factors for developing chronic ITP (older age, higher platelet count at diagnosis, and an insidious onset of symptoms) [20], and a proposed chronic disease prediction score was recently refined. However, no available treatment can currently alter the clinical course of ITP, even if prediction scores identify those at the highest risk for either bleeding or chronic disease. With regard to predicting response to therapy, studies found that response to treatment with IVIG is associated with Fc gamma receptor polymorphisms, as well as with other components of the adaptive or innate immune system [4,33,34]. Although currently not well validated or readily available, in the future, physicians may be able to use genotyping to recommend an individualized treatment approach. Perhaps, similar biologic and predictive data for other treatments could lend to a targeted treatment approach.

## 5. Options for Subsequent Therapies

The current consensus for the definition of refractoriness to therapy is reserved for those patients who have failed splenectomy [35]; however, as fewer patients are undergoing this procedure there is a need to reconsider this definition. While the majority of patients respond to one of the second-line therapies, approximately 10% do not. The majority of hematologists would agree that failing a second-line treatment should classify a patient as refractory, but this is not universal. As a result, an alternative definition of refractory was suggested and defined as a patient whose platelet counts do not respond to at least two treatments, there is no single medication to which they respond, and platelet counts are very low and accompanied by bleeding. However, before characterizing the disease as refractory, it may be necessary to conduct a careful reassessment of the patient, including an extended diagnostic workup to exclude known conditions which may mimic ITP.

Patients who are refractory to standard treatment may experience increased morbidity and mortality, as well as a significantly impaired HRQoL both from bleeding and from adverse effects of treatment [36]. As mentioned earlier, although life-threatening bleeding remains rare, the risk is not negligible, and can increase with the length of time with thrombocytopenia, with certain comorbidities or medications such as platelet-inhibitors and anticoagulants. Other concerns are frequent attendance at the hospital or clinic and anxiety around bruising and unpredictable platelet counts, which may increase in burden the longer a patient lives with ITP. For example, children with therapy-refractory ITP may need to reduce participation in activities and adults might need to reduce their work activity and socialization.

The indication for further therapy at this stage requires careful reassessment of the risk of bleeding, possible side effects of treatment, and a detailed discussion about the impact of the disease and treatment on HRQoL. Like in other stages of ITP, we lack validated, clinically useful tools for the assessment of patient-reported outcomes and bleeding risk. In addition, the effect and safety of most available therapies used for refractory ITP lack proper evaluation. Measures such as the use of tranexamic acid and hormonal management of menorrhagia may be sufficient for a cohort of patients. For others, however, further treatment is needed. It also may become important to reconsider the diagnosis at this stage.

In addition to the lack of a consensus on a definition for refractory ITP, the American Society of Hematology guidelines do not specifically address therapy beyond standard second-line due to a lack of robust evidence regarding management. They did comment that future research is needed to understand how new therapies should be positioned amongst existing second-line treatments, such as those discussed above [1]. The International Consensus Report recommends using therapies with robust evidence first [2]. Of these, fostamatinib, an oral medication that inhibits the Syk pathway, was recently licensed for use in patients with refractory ITP based on two randomized trials [37]. Pooled analysis of the two parallel phase 3 randomized trials showed 18% of patients achieved a stable response (platelet count >50 × 10^9^/L without rescue medication at four of six visits), with an overall response of 43% compared to 14% in the placebo arm [37]. Significant adverse effects to fostamatinib include diarrhea (in approximately 20%), hypertension, and abnormal liver function tests [37].

For patients who fail standard therapies, participation in clinical trials may be the next treatment option. Several new medications are currently undergoing evaluation in clinical trials. Bruton’s tyrosine kinase (BTK) inhibitor is about to complete a phase 1/2 trial (NCT03395210), where preliminary data from the ongoing study suggest responses of up to 40%. Additionally, several FcRn receptor inhibitors were developed and are currently in various stages of clinical development [38]. Also, new evidence of altered complement levels in patients with ITP led to phase 1 clinical trials of complement pathway inhibitors.

Outside of randomized trials, combination treatment gives the greatest probability of a platelet response for patients with refractory disease. Combination therapy could include combining two TPO-RAs or a TPO-RA with cyclosporine, cyclophosphamide, or azathioprine [36]. Hematopoietic stem cell transplantation was reported in small cohorts. Unfortunately, because hematopoietic stem cell transplantation is associated with high morbidity and mortality from bleeding and infection, it is an undesirable treatment approach for ITP and not currently recommended. Smaller studies of sirolimus are promising, with 73% of ITP patients achieving a complete response, and 78% of those patients achieving a complete response after only three months of treatment [39].

## 6. Conclusions and Future Directions

Patients with ITP face a complex set of challenges. ITP is a heterogeneous disease associated with significant morbidity not limited to bleeding. Due to the variability in its underlying pathobiology and natural history, management of ITP can be unpredictable, despite the availability of several therapies with different mechanisms of action. Once diagnosed, ITP patients experience a range of physical and emotional challenges as they seek to monitor their platelet counts, balance treatment side effects and manage the fear and frequent reality of relapse [40].

Despite recent advances in the management of ITP, many areas need further research. There is a need for brief, validated instruments that can both identify clinically significant patient-reported outcomes while also being feasible to incorporate into the routine clinical care of ITP. These instruments need to assess the impact of therapies on fatigue and HRQoL. Additionally, the potential negative impact of corticosteroids on patient’s lives needs greater attention. 

Patients state that achieving a healthy platelet count is important to them, however the majority of ITP therapies fail to induce remission. Consequently, there is a need to recognize the limitations of current therapies and evaluate new approaches. In light of recent guidelines recommending shorter courses of corticosteroids, ultimately, a first-line therapy with a quicker response and the ability to improve long-term remission would improve HRQoL and therefore be desirable. Currently, there is not sufficient data to recommend the addition of rituximab or TPO-RAs for first-line therapy for ITP; however, further data regarding cost, impact on HRQoL, long-term remission, and safety may provide insight into how to improve up-front management and integrate these therapies. 

## 7. Conclusions 

In conclusion, we should aim to develop a treatment model that reduces exposure to unnecessary medications by identifying patients more likely to benefit from treatment. Individualized treatment plans should be based on predicted or current bleeding severity, clinical course, HRQoL and the probability of biological markers of response. Further research is necessary to identify robust predictors of bleeding and chronicity of ITP and truly understand the patient experience. Furthermore, a validated screening tool that identifies clinically significant patient-reported outcomes in routine clinical practice would help both patients and physicians to effectively follow a patient’s HRQoL. Only then will we be able to best address a patient’s emotional and mental health needs in addition to treating the laboratory findings and physical symptoms of ITP.
